# Contextual and psychological factors influencing open defecation free status: an exploratory qualitative study in rural South Western Uganda

**DOI:** 10.1186/s12889-022-12759-z

**Published:** 2022-03-01

**Authors:** Moses Ntaro, Judith Owokuhaisa, John Bosco Isunju, Edgar Mulogo, John C. Ssempebwa

**Affiliations:** 1 Department of Disease Control and Environmental Health, School of Public Health, College of Health Sciences, Makerere University, P.O.Box 7072, Kampala, Uganda; 2 Department of Community Health, Faculty of Medicine, Mbarara University of Science and Technology, P.O. Box 1410, Mbarara, Uganda

**Keywords:** Open defecation free (ODF), Open defecation (OD), RANAS factors, Behavior, Contextual, Psychological

## Abstract

**Introduction:**

Achieving the Open defecation free (ODF) status remains a major challenge in Uganda, yet it contributes significantly to child health improvement. Literature on social, cultural and behavioral aspects that influence the ODF status in rural Uganda is limited. The study therefore, explored perceived factors influencing the ODF status in rural South Western Uganda.

**Methods:**

An exploratory study employing qualitative techniques and based on deductive analysis between month December 2020 and January 2021 was conducted. Seven Focus Group Discussions (FGDs and three Key Informant Interviews (KIs) were conducted in Kabale District, southwestern Uganda. Focus Group Discussion participants were mothers and fathers having children of 2 years and below while KIIs included local community leaders and health extension workers. Data was analyzed using a categorization matrix derived from the Risks, Attitudes, Norms, Abilities, and Self-regulation (RANAS) model which is comprised of contextual and psychological factors. Text was further categorized into high and low statements for attainment of ODF status.

**Results:**

The contextual factors influencing the Open Defecation Free status behavior included; farming activities far from home, financial constraints, rainy seasons, collapsible soft soils, and alcohol use. Psychological factors influencing ODF status included; perceived health risk for typhoid disease, low perceived severity for lack of ODF components, negative attitude of less value attached to ODF components, and a feeling of time wastage practicing ODF status behavior. The perception that the community has the ability to attain the ODF status was high. Although, the capability to maintain ODF was low when it comes to replacement of ODF component if stolen or destroyed.

**Conclusion:**

Open Defecation Free status is influenced by contextual and psychological factors. Therefore, it’s crucial for sanitation promotors to always identify such context specific factors in order to design sanitation and hygiene promotion interventions to address the ODF free status related challenges.

## Background

Globally, about 0.9 billion people still practice open defecation. Although there has been decrease in proportions of the population practicing open defecation in many regions of the world, the number of open defecators increased in sub-Saharan African countries by 16 million to 220 million [1]. Studies reveal that although governments have been spending on increasing latrine coverage for decades, rural open defecation remains high [2, 3]. More so research has also demonstrated that construction of more latrines does not result in reduction of oral-fecal diseases among children. However, open defecation-free status reduces such illnesses leading to improved child health [4]. For example, Abebe and Tucho [4] further established that the prevalence of diarrhea was much less in open defecation free (ODF) villages compared OD villages.

In a multi country study about ODF sustainability in four countries in Africa which included Uganda, a criteria for household-level ODF status certification was agreed upon. It required a household to have: no human feces in the vicinity, a latrine with a superstructure; with either a water seal for the water born systems or a latrine cover as a means of keeping flies from the pit, a hand washing facility with water and soap or ash, and evidence that a latrine and the hand washing facility were being used (e.g. latrine and handwashing facilities have a walkway path well-trodden on) [5]. Researchers in this study established that 8 % of households having a functioning latrine had visible signs of faeces around the house. Households with latrines having hand washing facilities with water and soap or ash, were 25% and those observed with lids covering the latrine drop hole were 19%. When all the five ODF status criteria were applied, the overall rate of households with ODF status across the study was 8% [5].

In Uganda, sanitation is still a challenge with 22.9% of the population practicing open defecation [6]. Also, 64% do not practice adequate hand washing (washing hands with water and soap) in the rural areas [6]. In addition, among 2/3 of the Districts in Uganda that receive the District Sanitation and Hygiene Conditional Grant annually from the government of Uganda, 63% of the villages in these districts have not attained the Defecation Free-ODF by 2019 [6, 7]. Rubaya and Buhara subcounties in Kabale District in South Western Uganda rank the lowest in sanitation status in Kabale district [8]. According to Ndorwa West Health Sub District annual health status report, 2018, 35% of the households in Rubaya subcounty did not have latrines and only 3 villages have received the Community Led Total Sanitation (CLTS) intervention of which 2 villages were declared Open defecation free.

Researchers have established that when households or communities are not living in an ODF environment, there are consequences for child mortality and development [9]. It’s under estimated that 2 million children die annually due to poor water, sanitation and hygiene diseases [10]. Mara [11] emphasized that seeking interventions to address OD should remain core to researchers and implementor. This is because OD has adverse health effects such as excreta-related infections and infestations which mostly affect the poor. More so, OD has been associated to psychosocial stress in women, stunting and missed school days among children as well as environmental pollution, income and productive time loss [12–14].

World Health Organization [15] argues that, considering the global agenda of eliminating OD practice by 2030 on the basis of the previous reduction rates, this goal remains ambitious. Mara [11] similarly, in his review paper concluded that elimination of OD by 2030 would not be realized. Unfortunately, while most of the regions registered significant OD reduction, 39 countries in sub-Saharan Africa had a 49 million population increase of open defecators during the millennial Development Goals (MDGs) period [16]. Although this was solely attributed to the population increase, there is a need to further explore other underlying factors to this negative trend. Abubakar [17] states that future research should focus on national level factors influencing OD if reduction and elimination of OD is to be accelerated in sub-Saharan Africa.

Odo and Mekonnen [18] established some of the factors that are associated with households that have handwashing facilities. These include; a better household wealth status, education status of the household head, having a radio and an improved latrine facility. In the same study, the authors concluded that if effective measures to increase handwashing are to be put in place, there is a need to understand contextual barriers such as existing policies, psychosocial factors and traditional norms. There are several contextual and behavioral factors that influence ODF status components with in different communities. These factors include structural, socio cultural, unpleasantness of the toilet, socio economic, locational, demographic and household characteristics factors [17, 19, 20]. Lopez, Berrocal [21] identified that social norms are important determinants for latrine use. Similarly, in a systematic review and meta-analysis study, privacy, better maintenance, cleanliness, facility type, accessibility, and newer latrines factors were commonly associated to higher latrine use, while poorer sanitation environment were associated to lower use [22].

To investigate contextual and psychological factors influencing open defecation free status, we adopted the combination of the Risks, Attitudes, Norms, Abilities, and Self-regulation (RANAS) model and the theory of triadic influence which elaborates social, physical and personal factors. The behavior change method was developed for evaluating behavior change interventions. The approach focuses at changing behavior factors of a given behavior in a population. This RANAS model framework combines behavior change theories of health action process approach and the theory of planned behavior [23]. The model is categorized among the psychological sanitation promotion approaches and focuses on development of interventions based on information about the population’s psychological determinants that are influencing a given behavior. Based on this information collected during the baseline phase, appropriate Behavior Change Techniques (BCTs) that target the identified factors are used in the intervention phase [24].

Therefore, the aim of this study is to investigate contextual and psychological factors influencing the ODF status using the RANAS model in order to gain an extended understanding of the perceived factors influencing ODF status in Rubaya and Bubara subcounties in Kabale.

## Methods

This study used an exploratory qualitative design. It employed Focus Group Discussions and Key Informant Interviews as data collection methods. Data was analyzed according to deductive qualitative content analysis technique [25].

### Study context

The study was conducted in two sub-counties of Rubaya and Buhara in Kabale district. These are among the sub counties with the poorest sanitation indicators in the district. Rubaya subcounty has a population of 12,797 males and 14, 930 females with a total of 6050 households. While Buhara subcounty has a population 12,300 males and 14,000 females with a total 5233 households. Both sub counties have 35% of their households not having latrines. In Rubaya 2 out of 81 villages attained ODF status and no village has attained ODF status in Buhara [26].

### Recruitment and participants

The maximum variation purposive sampling strategy [27] was used to ensure heterogeneity of community participants into the study. Participants for Focus Group Discussion (FDGs) were community members comprised of male heads of households and mothers with children aged below 5 years that were conveniently selected by the Village Health Team (VHT) coordinator in each village. However, to obtain the study participants, one parish from each of the sub counties was selected. The selected two parishes were those that ranked least in latrine coverage according to the Principal Health Inspector at the district health office. From these parishes, 4 villages, 2 from each parish were selected as the villages having the lowest latrine coverage. Finally, VHT coordinators selected 30 men and 37 women to participant in the 7 FGDs (3 for men and 4 for women) based on convenience sampling. In addition, a VHT coordinator, a Chairman Local Council 1 and a Health Assistant were selected for key information interviews (KIIs).

### Data collection

Interview guides were used to conduct the FGDs and KIIs in the months of December 2020 and January 2021. To ensure homogeneity, FGDs with men and those of women were conducted separately. Each FDG had 9 to 10 participants. The round or ‘U’ shaped seating was used during the discussion so that the facilitator together with the participants could see each other. Each interview began with an overarching question that aimed at encouraging narration: ‘Can you please tell us what a household should have in place to be considered as to have good sanitation and hygiene?’ To establish the group views on contextual factors influencing ODF, a similarly phrased question was used: ‘Can you please tell us the issues that are making your households not to attain the ODF status?’ and to explore group perceptions on RANAS psychological factors influencing ODF status in the community, also similarly phrased questions were employed such as: ‘Can you please tell us about the risks people think they can get from not attaining ODF status?’ Probing techniques, such as ‘What do you mean by saying…?’ and ‘Please tell me more about…’, were used. The FDGs interviews lasted between 57 and 117 min and were conducted at a place chosen by the VHT coordinators in consultation with the participants. Nearly, all interviews were conducted by the lead author in the local language (Rukiga), recorded, saved as audio files and transcribed directly to English verbatim by experienced qualitative research assistants. The lead author, who also speaks the same local language listened to the audios as he cross checked the transcripts before and during analysis.

### Data analysis

Qualitative content analysis approach was used as described primarily by Elo and Kyngäs [28] and Hsieh and Shannon [29]. The data analysis was conducted using a deductive approach and a directed content analysis using the comprehensive framework derived from the theory of triadic influence and the RANAS model. One of the major benefits of content analysis is its flexibility when it comes to research design and it allows the use of deductive or inductive depending on purpose of the research [28]. However, this study applied deductive content analysis; which is an approach appropriate when a priori theory exists about a phenomenon. Deductive content analysis is also useful in cases of retesting data in a new context such as in our study area where contextual and RANAS factors influencing ODF in a new social cultural environment were explored [28].

The analysis process began with the first author (NM) reading the transcripts to become familiar with them and gain an overall impression and sense of the texts. Later three of the co-authors (JS, EM, JBI) individually read a number (*n* = 2) of randomly chosen interviews whereas one of the co-authors (OJ) read all of them (*n* = 10), also to get an overall understanding of the material.

Subsequently, a structured categorization matrix [28] was developed for the RANAS MODEL’s five key factors and their sub-factors together with contextual factors. Next, more thorough reading of the transcripts was done and text corresponding to the contextual and RANAS factors categories in the matrix were highlighted with different colors, manually coded and transferred to the structured categorization matrix (Table 1). In the matrix, text was assessed and represented as high or low in relation to supporting an ODF environment. The analysis focused on exploring the contextual and RANAS factors influencing the ODF behavior. The first author (MN) took the lead in the analysis while the other authors (JBI, JO, EM and JS) evaluated and re-assessed the transferred texts into the different categories of the matrix.

**Table 1:** Categorization matrix

RANAS model categories	RANAS model sub categories	Description of the codes	Quotes	
			High statement for attaining ODF	Low statement for attaining ODF
**Risk factors**	**perceived health risks**	Diseases associated with individuals not living in an ODF environment	“Typhoid is the commonest but the problem with people in this village, when they feel ill, they just go to clinic and ask for drugs...without testing to know which disease they are suffering from.” (FGD, participant)	
	**Perceived vulnerability**	Children were perceived as the ones most affected by the ODF status		“We normally get cholera in children though they try and provide treatment but its common among children.” (FGD, participant) “For the old people, it’s not common for them to get diarrhea and when one gets it, he just thinks it’s what he has eaten or drank…. most say that maybe they have taken poor quality porridge or food and the stomach reacted like that” (FGD, participant)
	**Perceived severity**	**Low perceived severity if you live in a household without the ODF status**		“...most people are aware that malaria leads to death, most people slept under mosquito net but they don’t know the diseases which they get from toilet can kill them. They just get worms, go for medication and they become Okay..” (FGD, participant) “...if we had got someone dying after visiting latrine and leaving without covering, we should be covering them [using latrine covers to control flies] but we had never got such case in our area” (FGD, participant)
	**Factual knowledge**	**Knowledge on oral fecal transmission**	“It’s [diarrhea] caused by flies because when we are neighbors, they fly from one household to another. So that is why you find like 4 families in one village, their children are having diarrhea. Even the old people may be having diarrhea but they cannot disclose it.” (FGD, participant).	“...no one can say that the child got diarrhea because of dirtiness [feacal contamination]. They just say the child has got a stomach reaction [abdominal upset].” (FGD, participant)
		**Knowledge of ODF components**		“…So they don’t know the importance of those covers [pit latrine covers for flies control].” (FGD, participant) “People don’t wash hands because they don’t know the outcomes of it….. If they are aware that they can get diseases, I think they should be washing their hands.” (FGD, participant)
**Attitude factors**	**Positives and negatives: Costs/benefits for ODF behaviour**	**less importance attached to ODF components such as handwashing facility and latrine cover**		“Lack of seriousness is caused by the value attached to it [handwashing facility]. People thinking it’s a small thing and keep postponing doing it…” (FGD, participant) “...Even those who put the water jerricans for handwashing, many of them it’s like a decoration, washing is less…” When people hear that VHTs are coming, it’s when they use them but most of the time, those latrine covers are always in the corner [inside pit latrine’s floor corner].” (FGD, participant)
		**Wastage of resources put into attaining ODF status**		“...in this village, people have that feeling of wastage of time to have a clean household (ODF).” “..like apiece [a piece of soap] of 1000, there is one who calculates that the soap which he can keep putting at the latrine it can be spoiling my money...” (KII, participant)
		**Economic benefits attached to living in ODF environment**	“For sure such homes [homes with ODF components] don’t spend much due to sickness [faecal related diseases] and they end up developing but those who normally spent on these diseases [faeacal related] may end up in poverty.” (FGD, participant) “.....you save on the money and the time that you will be spending on the hospital, so you have time to do other things….” (FGD, participant)	
		**Health benefits attached to an ODF environment**	“….you are protecting your health and that of your family members. Secondary, they cannot get such diseases that are caused by dirtiness…..” (FGD, participant)	
	**Affective aspect**	**Peaceful feeling when living in an ODF household**	“…you feel having peace and your family cannot get disease” (FGD, participant)	
		**Proud for having living in an ODF household**	“..I found that I was one of those having a washing point…..... So that makes you feel comfortable in the heart and when people are passing around, you feel proud and even happy.” (FGD, participant)	
**Norm factors**	**descriptive norm (perceptions of ODF status by others)** **Injunctive norm (perceptions of ODF approved by important others).**	**Individuals living in households with ODF status were perceived to be more educated**	“They see that family [with ODF behaviour] as educated and they know what they are doing.” (FGD, participant)	
		**Individuals practicing ODF behavior such as handwashing after visiting latrine where perceived to be wasting time on less important things**		“...neighbors may think you are wasting time because they are spending some time without getting sick and they see you trying to put everything [handwashing facilities, latrine with latrine cover]] in place, they say you are wasting time.” (FGD, participant) “...Some take you to be a fool that what you are doing is nothing and yet you are doing cleanliness.” (FGD, participant)
		**Individuals demonstrating ODF behavior were perceived to show off/proud**		“People think it’s because he is rich. Others say you are proud.” (FGD, participant) “They see you as a pretender. Another person may choose to pick the jerrycan and throw it away simply because they look at that person as a pretender...a behavior of washing hands after visiting a latrine. It doesn’t look normal to many.” (FGD, participant)
		**Approval of ODF households by important others such as religious leaders**	“...when those people [important others] come to your home and find you are clean, they sit comfortably and even if you offer them food or a drink, they feel free to take it..” (FGD, participant)	
**Ability factors**	**Confidence to maintain ODF**	**High confidence**	“Most of the things can be done because we use our own hands to put them in place….... Even you may find you have like 3 to 6 jerrycans at home which are normally scattered in the compound not broken and you fail to put water in it and put it at the latrine. So we have the capacity to put such things in place..” (FGD, participant)	
		**Low confidence**		“When we talk of capacity [to have ODF components], me I see may be having soap, you cannot fail to have soap for washing or bathing and you get one to put on the toilet…” (FGD, participant)
	**Action knowledge for ODF items eg. latrine items and tippytape**	**High action knowledge**	“..people used to make latrine covers from banana fibers instead of timber because they knew how to do them…..So things are possible….they are knowledgeable”.	
		**Materials for ODF components**	“..the resources [items] are very available. Because Kabale is blessed with enough trees. So, one can’t use that as excuse for not building a latrine…. toilet covers can be made locally. And they know that they have the resources to construct them.” (KII, participant)	
**Self-regulation factors**	**capability to plan and self-monitor including managing contradictory goals and distractions**	**Discouragement related to loss or destruction of an ODF component**		“...but they kept stealing the soap. I got discouraged and leave them.” (FGD, participant)
		**Commitment to replacing destroyed or missing ODF components such as handwashing facility**	“I keep replacing it because I know its importance”	
**Contextual factors**	Social context	Farming activities away from home where there are no latrines		“...I may be having my latrine here and wanted to go and cut my trees up the hill there. So, when I go to cut them and I am spending the whole day there, do you think I can come to defecate and go back? I just defecate there because you cannot put toilet in every bush”. (FGD, participant) “.. When we go digging, we tend to use the gardens for our defecation.” (FGD, participant)
		low-household income level		“It also depends on the income of someone. I cannot fail to have one [piece of soap] for bathing or washing clothes and buy the one to put on the latrine..... So, I think if there is a possibility of an alternative like detergents that dissolve in water and is given by government to people, this can help everyone to comply.” (FGD, participant)
		**Ash use replacing latrine covers for controlling flies**		“..Bakiga are intelligent, when the latrine start smelling and flies come out, they pour ash inside and the smell goes even the flies.” (FGD, participant)
		**Gender roles which lead men to be less involved in ensuring that their households are ODF**		“For us Bakiga, we believe that a man is the head of the family and depending on our responsibilities, when am home with my wife, I tell her to clean up the place…....according to Bakiga culture, such responsibilities [removing children feaces from compound] belong to wives and in most cases, they are the ones to take care of the children”. (FGD, participant) “The man digs [pit latrine hole] it after he builds it when he finishes it, issues of water [hand washing water] and the rest are for the woman; you find the woman saying that you leave these things for me”. (KII participant)
		**community by-laws**	“...when leaders pass around and find for example a person with no latrine or its not clean, they fine you” (FGD, participant) “...The ones [latrines] we find full, we put them down and give you the deadline for putting up another one and on top of that, they fine you with shs. 20,000/= [approximately USD 6]”. (FGD, participant)	“The laws at village level were there but with time, those laws lost value...”. (FGD, participant) “...These days we are somehow helped by the VHTs, but even if they get you and take you to prison, even those who take you, they find you home by the time they return meaning, the laws are not strengthen and the penalties are not there”. (FGD, participant) “...Even if you arrest someone like now for not having a toilet, he would be home again in a few minutes as the politicians will say that you are spoiling their votes.” (KII, participant)
		**Information access**	“...a person who is sensitized there is how he can slightly change, you find that he is not like the other one because there is when I come with the health people, they come and say that let us go and inspect [household sanitation inspection], when they do the inspection, you find before the end of the month you find that there is a difference, you find that there is something that has changed [towards attaining ODF components].” (KII, participant)	“We are not informed and that’s why most don’t wash hands after visiting latrine. In case we have leaders to educate us, like they blow whistle [calling community gathering], gather people and tell them what they are supposed to do, I think they can do ....” (FGD, participant) “...I met a man from those hills who found a jerrycan of water and soap [handwashing facility] at the toilet and asked me the importance of them. He was not informed of its importance, so others fail to do them because they are not aware of them or are not taught.” (FGD, participant)
		**Shared latrines**	“Since we have nearby households in this area of Mushenyi like less than 10 m apart or even less and since we coordinate with each other, toilets are not locked and one can use for the neighbor…”	“..you find an extended homestead with about 4 households all using the same latrine. So, at times it may happen when its dirty [soiled] and none is willing to clean it. All end up abandoning it.” (FGD, participant)
	Physical context	Water access		“...someone from up the hill fetches water from valley and as I talk is still in the garden up to 4:00 pm. From there he will come with one jerrycan to fetch water which is to be used for drinking and cooking then after goes back to fetch for bathing. So, it becomes hard for this person to get like 3 l [water] to put it the small jerrycan on the latrine...” (FGD, Participant)
		Temporary latrines		“When you are leaving in a place where there are ants in low areas or in places which are soft and you build with wood which they normally use here, latrines reach time and fall....” (FGD, participant) “...when it’s at night, I cannot also move to look for toilet for fear that it might fall on me when no one knows. So, I also defecate on the roadside.” (FGD, participant)
		Rainy seasons		“In this area we are living in is water logged place and in rainy season, some latrines fall down and it take some time to be reconstructed, may be after when the rainy season is over. So, during that period, even if they say they have toilets but they are not in good conditions.” (FGD, participant)
		Small land size		“...you might find someone had a small plot and after construction of a house, there is nowhere to put the latrine.” (FGD, participant)
		Soft collapsible soils		“...I was telling you about our soils on the mountains, a person can be staying on the mountain, he builds his good latrine after 1 year when the rain comes it washes everything away….this year, the latrines that have fallen are not below 50 and all of them fell within 2 months.” (KII, participant)
	Personal context	Young children contributing to failure open defecation practice		“In our village….., the child defecates wherever they are, either in the house or compound and its up to parents/ caretakers….3 years and below, they defecate wherever they are. So, when you are a dirty person, you find feces everywhere in the house, veranda, compound etc. or you find [children feaces] for 3 or 4 days still there”. (FGD, participant)
		**Alcohol abuse contributing to open defecation**		“... drunkards who defecate in gardens and along the road because they have no time to look for toilet and they use [defecate] wherever they see and go.” (FGD, participant)
		**Vulnerable individual such as older persons unable to put in place the ODF components**		“…widows are the ones who have no latrines.” (FGD, participant) “...aged ladies who cannot afford to have such things [latrine, handwashing facility, latrine covers] and cannot get someone to build them toilets.” (FGD, participant)

## Results

We conducted seven FGDs and three KIIs. Thirty men and 37 women participated in the seven FGDs (three for men and four for women). In addition, a VHT coordinator, a Chairman Local Council 1 (political head of the village) and a Health Assistant participated in the KIIs. The socio demographics of the participants are detailed below (Table 2).

**Table 2:** Socio demographics characteristics of the participants

Characteristics	Participants (*n* = 67)
Gender	
Male	30 (44.8%)
Female	37 (55.2%)
Age (years)	
Mean	32
Range	20–63
Religion	
Catholic	43 (64.2%)
Pentecostal	1 (1.5%)
Protestants	23 (34.3)
Marital status	
Married	65 (97.0%)
Widowed	1 (1.5%)
Not married	1 (1.5%)
Education level	
Primary	44 (66.7%)
Secondary	21 (32.8%)
Tertiary	1 (1.5%)
Economic status	
Peasant farmer	48 (71.6%)
Self employed	14 (20.9%)
Employed	5 (7.5%)

Most 97.0% of the participants were married and had attained the primary level of education (66.7%). More so most (71.6%) of the study participants were peasants growing mainly food for household consumption.

### Deductive analysis

The deductive analysis revealed that the transcribed text strongly reflected three of five categorisation matrices, i.e. risk, attitude and norm factors representing the RANAS MODEL in addition to the contextual aspects [30]. The elements and sub-elements of the model were represented by texts reflecting respondent’s perception expressed on a given element based on high to low statement continuum as reflected (Table 1).

The deductive analysis showed statements reflecting the high end of the continuum in relation to health risk associated with living in households that are not ODF. Statements presenting attitude factors especially the negatives such as time wastage associated with households attaining the ODF status represented the low end of the continuum. At the high end of the continuum, statements concerning the health and economic benefits perceived to be enjoyed by the individuals living in ODF households were also aspects of the attitude factors expressed (Table 1). The element norm factors were denoted by statements reflecting descriptive norms of attaching no value to putting in place and using ODF components such as handwashing facility and latrine covers. This represented the low end of the continuum respondents perceived themselves to be viewed as fools if they are seen by other community members putting in effort to have and maintain an ODF household and environment. At the high end of the continuum, statements concerning injunctive norms where respondents expressed that important others in the community appreciate and feel conformable in households which are ODF. The element ability factors were denoted by statements reflecting communities confidence to use locally available materials to put in place most of the components for a household to attain the ODF status. Such statements represented the high end of the continuum. However, statements where respondents felt that if a household is failing to have money to buy a piece of soap for bathing, then how can they have one for the handwashing station represented the low end of the continuum. Finally, contextual factors were denoted by statements reflecting low household income, farming activities far away from latrine facilities, low levels of awareness when it comes ODF importance, difficulties in accessing water, hilly terrain affecting stability of latrines, heavy rains washing away latrines and alcoholism leading to OD behavior. These represented the low end of the continuum. At the high end of the continuum, statements reflecting law enforcement and penalties compelling some community members to construct latrines were expressed.

### Contextual factors influencing open defecation free status

#### Social context

**Farming far from home** was identified as a driver for open defecation. Most of the community members grow crops and graze animals uphill far from their homes. Mostly, women and older children do the digging and weeding of their farm gardens. The farmlands for most households are deemed to be far and villagers normally leave very early and return home shortly before it gets dark. Men mostly do bush clearing and tree cutting in addition to grazing of livestock which takes place in the hills and wetlands. Since there are no latrine facilities located in grazing and farming areas, villagers resort to open defecation. “...I may be having my latrine here and wanted to go and cut my trees up the hill there. So, when I go to cut them and I am spending the whole day there, do you think I can come to defecate and go back? I just defecate there because you cannot put toilet in every bush”. (FGD, participant).

##### Financial constraints

Our study revealed that low household income level was among the factors influencing ODF. For example, some participates lamented that, “if we are failing to afford a piece of soap for bathing and washing clothes, how can we have soap to put at the handwashing station?” (FGD, participant). Indeed, most (71.6%) of the study participants were peasant farmers growing mostly food crops for home consumption (Table 2).

##### Gender roles

According to the finding of this study, men are less involved in maintaining ODF environment at the household level. Apart from building a latrine if able, all other aspects of removing children’s feces around the household, putting and maintaining a handwashing station, providing a fly trap cover for the latrine and cleaning soiled latrines are seen, as responsibilities of women and children. A KII participant said, “The man digs [pit latrine hole] and after he builds it when he finishes it, issues of water [hand washing water] and the rest are for the woman; you find the woman saying that you leave these things for me”.

##### By-laws

Our study found that there are existing laws and policies frameworks to support local leaders and sanitation implementers to enforce households to acquire a latrine. In a FGD a participant mentioned that heads of the households who do not have latrines at home are fined about six dollars and given a specific period when to complete latrine construction. On the other hand, the study findings reveal that weak enforcement of the existing by-laws has contributed significantly to OD. It was expressed that local political leaders restrain the sanitation implementer from arresting and fining individuals who do not have latrines for fear of losing their popularity. “...Even if you arrest someone like now for not having a toilet, he would be home again in a few minutes as the politicians will say that you are spoiling their votes”, said a KII, participant.

##### Information access

Lack of information about the necessity of a maintaining an ODF environment identified also as a cause of OD in the study area. The finding reveal that some community members have no access to information pertaining to importance ODF components. Some participants were merely, not having ODF households because they did not have information about it. For example, they urged that if someone had informed them about the importance of handwashing with soap, using a fly trap to cover the latrine drop hole and consistent use of latrine, they would have adopted the behavior. A study participant during the FGD said, “...I met a man from those hills who found a jerrycan of water and soap [handwashing facility] at the toilet and asked me the importance of them. He was not informed of its importance, so others fail to do them because they are not aware of them or are not taught”.

#### Physical context

##### Water access

Fetching water far from home was expressed as a key factor affecting the handwashing component of the ODF status. Water sources commonly located in valleys were perceived to be far from homes especially for those living up in the hills. Study participants expressed low statements to attaining ODF because of the difficulty in collecting water. They mentioned that it was not possible for individuals to fetch water for cooking and bathing and spare some for the handwashing station. A FGD participant mentioned, “...someone from up the hill fetches water from valley and as I talk is still in the garden up to 4:00pm. From there he will come with one jerrycan to fetch water which is to be used for drinking and cooking then after goes back to fetch for bathing. So, it becomes hard for this person to get like 3litres [water] to put it the small jerrycan on the latrine...”.

#### Personal context

##### Age of children

In the study area children aged 3 to 4 years are not allowed to use the pit latrines for fear of them falling into the pit. Children are shown areas around the compound or close to the latrine were to defecate in the open. The practice is that when the mother returns in the evening from the farming activities, she removes the feces and disposal it appropriately. Unfortunately, during most of the FDGs participants argued that some mothers are not able to remove children feces daily, hence hindering the attainment of the ODF status. “In our village….., the child defecates wherever they are, either in the house or compound and its up to parents/ caretakers….3 years and below, they defecate wherever they are. So, when you are a dirty person, you find feces everywhere in the house, veranda, compound etc or you find [children feaces] for 3 or 4 days still there”, said a FGD participant.

#### Psychological factors

##### Risk factors

Our study reveals that the study participants perceive the health risk of contracting diseases such as typhoid if they do not live in an ODF environment. However, in most of the FDGs participants highlighted that it’s children who are vulnerable to this risk. They further argued that adults do not associate any diarrheal disease they suffer to fecal contamination as a result of OD but to consumption of spoiled food or drink. This very low perceived vulnerability makes them feel not to be at risk and hence no reason to put efforts to attain an ODF status. More so, they had low perceived severity concerning diarrheal diseases and did not envisage the dangers of not washing hands, covering latrines and defecating in the open. “...if we had got someone dying after visiting latrine and leaving without covering, we should be covering them [using latrine covers to control flies] but we had never got such case in our area”, mentioned a FGD, participant.

##### Norm factors

The descriptive norms expressed during FDGs include statements showing that other community members perceived efforts to attain the ODF status to be waste of time. For example, there was an expression in most of FDGs that individuals seen regularly washing handing after visiting latrine are pretenders and showing off. “They see you as a pretender. Another person may choose to pick the jerrycan and throw it away simply because they look at that person as a pretender...a behavior of washing hands after visiting a latrine. It doesn’t look normal to many.”, said a FGD participant.

##### Ability factors

In our study, high statements depicting opinions of confidence to locally get ODF components such as small empty jerrycans for making handwashing stations, trees for providing poles to construct latrines, and banana plants fibers for making latrine covers were stated. On the other hand, our findings reveal low statements on ability such as low confidence in availing soap at the handwashing station. The positive and negative opinions concerning ability factors are as result of having both doers and non-doers of the behavior in the FGDs. “Most of the things can be done because we use our own hands to put them in place….... Even you may find you have like 3 to 6 jerrycans at home which are normally scattered in the compound not broken and you fail to put water in it and put it at the latrine. So, we have the capacity to put such things in place...”, mentioned a FGD participant.

## Discussion

This research aimed to investigate contextual and psychological factors influencing ODF status using the RANAS framework. The results of our deductive analysis fit well in the matrix we developed (Table 1).

### The contextual and psychological factors influencing ODF status

The researchers of this study used the RANAS factors model to established factors for open defecation-free status. The factors that emerged from qualitative deductive content analysis were grouped into contextual comprising of social, physical and personal factors and psychological comprising of risk, attitude, norm, ability and self-regulation factors.

### Social context

Farming far from home as a driver for open defecation. Since there are no latrines and handwashing facilities near the farmlands, individuals’ resort to open defecation in the gardens, bushes and in tree plantations. A study in Kenya also identified the farming occupation especially of men to be a key factor for OD in Lowdar area. Men revealed that they cannot come back to access a latrine so they defecate anywhere [31]. O’Reilly, Dhanju [32] established also in a study conducted in Uttarakhand, India that most men and women who defecate in the open do it because of farming and livelihood activities. It is important for sanitation promoter to be aware of this factor and focus on helping the farming communities move up on the sanitation ladders. More so, research focused on good sanitation options for farmers farming far from home are required.

Low household income level factor influencing ODF. Households with less income have less purchasing power even for buying small items such as a piece of soap (ie. a bar of washing soap cut into smaller pieces) which is less than half a dollar. Such financial status has a negative impact on households’ capacity to maintain ODF status. A study in Ghana to identify reasons for OD, revealed that respondents had serious income challenges with many lamenting of debt accruing from borrowing money for other things, such as food. Therefore, they did not have money for putting up latrine facilities. Other studies have also established that households with poor economic status are less likely to have latrines compared to those that are wealthier [2, 33]. Similarly, in another study identifying reasons for ODF slippage, it was revealed that lack of money to maintain or build permanent latrine facilities was among the key factors [4]. This underpins the importance of integrating sectors for household income improvement when designing sanitation and hygiene promotion strategies to address challenges for attaining ODF households.

Our study reveals that men are less involved in maintaining ODF environment at the household level. This poses a challenge to women who are already overburdened with gardening, collecting water, cooking and other home hygiene issues such as washing clothes and caring for the young children. Hence, ODF status is often compromised as handwashing stations and fly trap covers get ignored while children’s feces littered around the home is also unattended. In a similar context of identifying barriers leading to OD in Uttar Pradesh, India, men where not concerned about building latrines and insisted that there was nothing wrong with the practice of defecating in open fields. In the same study, researchers concluded that there is a need for sanitation programmes to involve both men and women [34]. Therefore, it’s important that hygiene and sanitation interventions be designed to involve men and women at all levels of project implementation.

More so, because of weak enforcement of existing laws and policies frameworks aimed ensuring that each household gets a latrine. Some community members do not take the issue of ODF serious. In similar study in Wa municipality in Ghana, non-enforcement of sanitation by-laws was identified as one of the causes of open defecation [17]. Therefore, creation of awareness of existing by-laws focusing on creating ODF environment, and involving community members in their implementation is likely to increase their enforcement.

Lack of information about the necessity of a maintaining an ODF environment was identified also as a cause of OD in the study area. This could be associated with the low level of education that was the case in our study area [17]. Implying that they had limited knowledge on oral-fecal transmission routes of diseases. Therefore, a participatory sanitation and hygiene intervention would increase the knowledge about sanitation and hygiene among the community members in the study area.

Shared latrines were considered to contribute to failure in attaining the ODF status. It is urged that as neighbors share latrines and soil them, they remain dirty and the users slip back to OD. Abebe and Tucho [4], in their systematic review paper established that 19 % of selected studies revealed that latrine sharing contributes to ODF slippage. They concluded that sharers decide to continue OD until they construct their own latrines. In another related report by the Water Supply and Sanitation Collaborative Council (WSSCC), individuals from poor households that were sharing latrines with neighbors often resorted to defecation in the open [35]. Therefore, participatory sanitation and hygiene campaign such as community led total sanitation can lead to increased household latrine ownership and usage [36].

### Physical context

Fetching water far from home is a key factor affecting the handwashing component of the ODF status in the study area. Among predictors for handwashing, water access was found to be significantly associated to increased handwashing behavior [37]. More so, in another study that measured water for household consumption, there was a more likelihood for households to practice handwashing when the amount of available to the household exceeded 7.5 l [38]. Therefore, consideration to address the water access challenge is paramount in promoting the handwashing component of ODF status.

Rainy seasons leading into water logging in villages continues to be a key driver hindering ODF status in the study area. Some latrines are inundated during the rainy seasons and household members resort to OD. This finding is in agreement with similar study in India that showed non-use of water logged latrines during the monsoon floods [39]. The latrine situation is further exacerbated by soft soils resulting into latrine collapse during rainy seasons. A study conducted in Mozambique showed that 60% of individuals whose latrines collapsed did not rebuild their latrines and people who built their latrines on sandy soil slipped back to open defecation following their latrines [40–42].

Lastly, latrines constructed with low-cost temporary materials were also reported to be susceptible to collapsing due to destruction by ants. Commonly, the population in the study area uses untreated wooden poles, mud and wattle to construct the sub structure slab and supper structure, often using dried grass for roofing latrines. When the latrines collapse, it takes some months before there are rebuilt, meanwhile the household members often resort to OD practices or using the neighbor’s latrine. A study in India established that the use of low-cost material to construct latrine facilities contributes to ODF slippage [43]. Hueso and Bell [44] indicated that permanent toilets have the ability to sustain open defecation-free status for a long time, while a short-lived latrine result into individuals abandoning them or preferring open defecation. Therefore, promoting construction of permanent latrines that more resistant to adverse environmental challenges should prioritized in the sanitation improvement campaign.

### Personal context

Children aged 3 to 4 years are not allowed to use the pit latrines for fear of them falling into the pit. Children are shown areas around the compound or close to the latrine were to defecate in the open. The practice is that when the mother returns in the evening from the farming activities, she removes the feces and disposal it appropriately. Unfortunately, during most of the FDGs participants argued that some mothers are not able to remove children feces daily, hence hindering the ODF status. In another study in Guwahati, India, small children in the household were left to defecate in the open since the women were busy with house hold chores in the morning [45]. Another, factor mentioned to be contributing to failure to attain ODF was alcohol use. It was mentioned in almost all the FDGs that drunkards were the main open defecators on paths and major village roads. This finding is similar to the one in rural south India where alcohol use was found to be higher among individuals who prefer defecating in the open.

### Psychological factors

Study participants perceived the health risk of contracting diseases such as typhoid if they did not live in an ODF environment. In another study investigating the CLTS intervention on rebuilding latrines which collapse Mosler, Mosch [40] relieved that persons who perceived that they behavior of open defecation is likely to affect the health of others were more likely to rebuild the latrine. Therefore, interventions such as CLTS that increases the perceived risk for individuals practicing OD would be ideal. However, the attitude of attaching less value on ODF behavior in the study areas makes residents vulnerable to its consequences. This is because some individuals are likely not to practice the ODF behavior and therefore at high risk contracting infectious diseases that thrive under poor environmental sanitation conditions [17].

More so, attaining ODF status through regular handwashing with soap and covering the latrine drop holes with covers was perceived to be resource wasting. However, the affective factor of feeling proud for having all the ODF status components and opinions expressed that households with an ODF status spend less on medical expenses treating fecal related diseases can be used as a basis to trigger behavior change. This finding is in agreement with Tumwebaze and Mosler [46] who established that the RANAS affective factor was significantly associated to the latrine cleaning behavior among slum dwellers sharing latrine facilities. In the same study they argued that there is a need for persuasive approaches emphasizing the health benefits for performing the required behavior if it’s to be adopted. Therefore, a similar sanitation and hygiene promotion intervention stressing the good health attributes for living in an ODF environment would encourage the adoption of ODF components and behavior.

The descriptive norms expressed such as efforts to attain the ODF status perceived as a waste of time and regular washing handing after visiting latrine viewed as showing off have the potential for hindering the adoption of the ODF status in the study area. A related study by Mulopo, Kalinda [47], established a significant difference on the descriptive norm scale between those who were using safe water sources and those who were not. The researchers argued that there is need for community members to make public commitment to collect water from safe sources as well get support from community leaders to promote both descriptive and injunctive norms.

Lastly, the ability factor expressed such as low confidence in availing soap at the handwashing station was also hindering attainment of the ODF status. This is related too to the finding by (Mulopo, Kalinda [47]) who found that the low confidence ability in consistence use of safe water was contributing to the practice of using unsafe water sources. In the same study, it was denoted that the reason why there was low confidence is that there are limited safe water sources in the village. This is similar in our study because the reason for low confidence of having soap at the handwashing station was affordability. Integrated efforts to improve hygiene together with household livelihoods is crucial in addressing the handwashing challenge.

### Limitations

Since the study was purely qualitative, we were not able to quantify the doers and non-doers of the ODF related practices and behavior. The focus was on perceptions and opinions which we cannot rely on to make conclusions on the ODF status of the study area. More so, the identified factors in this study are specific to setting and other similar setting and cannot be generalized to all settings. Future research to quantify the ODF status and its associated factors should be prioritized so as to further understand the contextual and behavioral factors influencing ODF for purposes of designing focused Public Health Interventions.

## Conclusions

We explored contextual and psychological factors influencing people’s behavior with respect to ODF behavior and practices. The key factors were farming activities far from home, financial constraints, gender roles, by-laws, information access, shared latrines, water access, rainy seasons, collapsible soft soils, land size as key contextual factors for ODF status and psychological factors such as perceived health risks, ODF benefits, affective beliefs, descriptive norms of less value attached to ODF components, injunctive norms of pride for having all ODF components and low and high confidence as important factors for ODF status in the study area. We propose some recommendation to focus on in order to support such communities to attain the ODF status;

- It is important for sanitation promoter to be aware of open defecation practice due to farming activities and focus on helping the farming communities move up on the sanitation ladders,
- integrating sectors for household income improvement when designing sanitation and hygiene promotion strategies to address challenges for attaining ODF households,
- promoting construction of permanent latrines that are more resistant to adverse environmental challenges should be prioritized in the sanitation improvement campaign,
- ecological sanitation latrines can be promoted among households with small land sizes,
- implementation of psychological hygiene and sanitation interventions such as community led total sanitation (CLTS) that increase the perceived risk and influence attitudes and norms for individuals practicing OD.

More so, research focused on good sanitation options for farmers farming far from home and establishing the ODF status together with quantifying the influence each factor has on ODF status is required.

## Data Availability

Due to restrictions set by the Makerere University, College of Health Science High Degrees Research and Ethics Committee, data are available upon request by contacting the corresponding author.

## Abbreviations

BCTs: Behavior Change Techniques
CLTS: Community Led Total Sanitation
FGDs: Focus Group Discussions
KIIs: Key Informant Interviews
OD: Open defecation
ODF: Open defecation free
RANAS: Risks, Attitudes, Norms, Abilities, and Self-regulation
VHT: Village Health Team
WSSCC: Water Supply and Sanitation Collaborative Council

## References

[1] World Health Organization W (2017). Progress on drinking water, sanitation and hygiene: 2017 update and SDG baselines.

[2] Adhikari R, Ghimire S (2020). Open defecation free: where do we need to focus?. Health Prospect.

[3] Coffey D, Gupta A, Hathi P, Khurana N, Spears D, Srivastav N (2014). Revealed preference for open defecation. Econ Polit Wkly.

[4] Abebe TA, Tucho GT (2020). Open defecation-free slippage and its associated factors in Ethiopia: a systematic review. Syst Rev.

[5] Tyndale-Biscoe P, Bond M, Kidd R. ODF sustainability study: FH Designs Australia: PLAN International; 2013. p. 1–181.

[6] MWE MoWaEGoU (2019). Water and environment sector performance report 2019.

[7] Rakotomanana H, Komakech JJ, Walters CN, Stoecker BJ (2020). The WHO and UNICEF Joint Monitoring Programme (JMP) indicators for water supply, sanitation and hygiene and their association with linear growth in children 6 to 23 months in East Africa. Int J Environ Res Public Health.

[8] Uganda Bureau of Statistics U. The national population and housing census 2014 – area specific profile series. Kampala: Government of Uganda; 2017.

[9] Vyas S, Srivastav N, Mary D, Goel N, Srinivasan S, Tannirkulam A (2019). Measuring open defecation in India using survey questions: evidence from a randomised survey experiment. BMJ Open.

[10] Fuente D, Allaire M, Jeuland M, Whittington D (2020). Forecasts of mortality and economic losses from poor water and sanitation in sub-Saharan Africa. PLoS One.

[11] Mara D (2017). The elimination of open defecation and its adverse health effects: a moral imperative for governments and development professionals. J Water Sanit Hyg Dev.

[12] Jewitt S (2011). Geographies of shit: spatial and temporal variations in attitudes towards human waste. Prog Hum Geogr.

[13] Kerstens S, Spiller M, Leusbrock I, Zeeman G (2016). A new approach to nationwide sanitation planning for developing countries: case study of Indonesia. Sci Total Environ.

[14] Sahoo KC, Hulland KR, Caruso BA, Swain R, Freeman MC, Panigrahi P (2015). Sanitation-related psychosocial stress: a grounded theory study of women across the life-course in Odisha, India. Soc Sci Med.

[15] Organization WH. Progress on household drinking water, sanitation and hygiene 2000-2017: special focus on inequalities: World Health Organization; 2019.

[16] Organization WH. National systems to support drinking-water: sanitation and hygiene: global status report 2019: UN-Water global analysis and assessment of sanitation and drinking-water: GLAAS 2019 report. 2019.

[17] Abubakar IR. Exploring the determinants of open defecation in Nigeria using demographic and health survey data. Sci Total Environ. 2018;637:1455–65. 10.1016/j.scitotenv.2018.05.104 29801238

[18] Odo DB, Mekonnen AG (2021). Availability and factors influencing community level handwashing facility in Ethiopia: implication for prevention of infectious diseases. PLoS One.

[19] Tessema RA (2017). Assessment of the implementation of community-led total sanitation, hygiene, and associated factors in Diretiyara district, eastern Ethiopia. PLoS One.

[20] Yogananth N, Bhatnagar T (2018). Prevalence of open defecation among households with toilets and associated factors in rural South India: an analytical cross-sectional study. Trans R Soc Trop Med Hyg.

[21] Lopez VK, Berrocal VJ, Angulo BC, Ram PK, Trostle J, Eisenberg JN (2019). Determinants of latrine use behavior: the psychosocial proxies of individual-level defecation practices in rural coastal Ecuador. Am J Trop Med Hyg.

[22] Garn JV, Sclar GD, Freeman MC, Penakalapati G, Alexander KT, Brooks P (2017). The impact of sanitation interventions on latrine coverage and latrine use: a systematic review and meta-analysis. Int J Hyg Environ Health.

[23] Schwarzer R (2008). Modeling health behavior change: how to predict and modify the adoption and maintenance of health behaviors. Appl Psychol.

[24] Mosler H, Contzen N (2016). Systematic behavior change in water, sanitation and hygiene. A practical guide using the RANAS approach.

[25] Bengtsson M (2016). How to plan and perform a qualitative study using content analysis. NursingPlus Open.

[26] GoU GoU (2018). Ministry of Health Environmental Health Division Rubaya subcounty sanitation report, Kabale.

[27] Etikan I, Musa SA, Alkassim RS (2016). Comparison of convenience sampling and purposive sampling. Am J Theor Appl Stat.

[28] Elo S, Kyngäs H (2008). The qualitative content analysis process. J Adv Nurs.

[29] Hsieh H-F, Shannon SE (2005). Three approaches to qualitative content analysis. Qual Health Res.

[30] Mosler H-J (2012). A systematic approach to behavior change interventions for the water and sanitation sector in developing countries: a conceptual model, a review, and a guideline. Int J Environ Health Res.

[31] Busienei PJ, Ogendi GM, Mokua MA (2019). Open defecation practices in Lodwar, Kenya: a mixed-methods research. Environ Health Insights.

[32] O’Reilly K, Dhanju R, Goel A (2017). Exploring “the remote” and “the rural”: open defecation and latrine use in Uttarakhand, India. World Dev.

[33] Osumanu IK, Kosoe EA, Ategeeng F. Determinants of open defecation in the Wa municipality of Ghana: empirical findings highlighting sociocultural and economic dynamics among households. J Environ Public Health. 2019;2019. 10.1155/2019/3075840 PMC6378051 30853996

[34] Khanna T, Das M (2016). Why gender matters in the solution towards safe sanitation? Reflections from rural India. Glob Public Health.

[35] House S, Ferron S, Cavill S. Scoping and diagnosis of the Global Sanitation Fund’s approach to Equality and Non-Discrimination (EQND): Water Supply and Sanitation Collaborative Council; 2017. p. 34. http://wsscc.org/wp-content/uploads/2017/08/GSF-EQND-Study-EN.pdf

[36] Venkataramanan V, Crocker J, Karon A, Bartram J. Community-led total sanitation: a mixed-methods systematic review of evidence and its quality. Environ Health Perspect. 2018;126(2):026001. 10.1289/EHP1965 PMC6066338 29398655

[37] Schmidt WP, Aunger R, Coombes Y, Maina PM, Matiko CN, Biran A (2009). Determinants of handwashing practices in Kenya: the role of media exposure, poverty and infrastructure. Tropical Med Int Health.

[38] Seimetz E, Boyayo A-M, Mosler H-J (2016). The influence of contextual and psychosocial factors on handwashing. Am J Trop Med Hyg.

[39] Routray P, Schmidt W-P, Boisson S, Clasen T, Jenkins MW (2015). Socio-cultural and behavioural factors constraining latrine adoption in rural coastal Odisha: an exploratory qualitative study. BMC Public Health.

[40] Mosler H-J, Mosch S, Harter M (2018). Is community-led total sanitation connected to the rebuilding of latrines? Quantitative evidence from Mozambique. PLoS One.

[41] Munkhondia T. On the road to sustainable sanitation: an overview of practices and lessons learned from a sanitation programme in Malawi. Waterlines. 2013;32(1):50–7.

[42] Sugden S (2003). One step closer to sustainable sanitation: the experiences of an eco-sanitation project in Malawi.

[43] Bongartz P, Vernon N, Fox J (2016). Sustainable sanitation for all: experiences, challenges and innovations: practical action.

[44] Hueso A, Bell B (2013). An untold story of policy failure: the total sanitation campaign in India. Water Policy.

[45] Jewitt S, Mahanta A, Gaur K (2018). Sanitation sustainability, seasonality and stacking: improved facilities for how long, where and whom?. Geogr J.

[46] Tumwebaze IK, Mosler H-J (2014). Shared toilet users’ collective cleaning and determinant factors in Kampala slums, Uganda. BMC Public Health.

[47] Mulopo C, Kalinda C, Chimbari MJ (2020). Contextual and psychosocial factors influencing the use of safe water sources: a case of Madeya Village, uMkhanyakude District, South Africa. Int J Environ Res Public Health.

